# *CTC-537E7.3* as a Liver-Specific Biomarker for Hepatocellular Carcinoma: Diagnostic and Prognostic Implications

**DOI:** 10.3390/cimb47070563

**Published:** 2025-07-18

**Authors:** Hyung Seok Kim, Se Ha Jang, Geum Ok Baek, Moon Gyeong Yoon, Jaewon Shim, Ji Eun Han, Soon Sun Kim, Jae Youn Cheong, Jung Woo Eun

**Affiliations:** 1 Department of Biochemistry, Kosin University College of Medicine, Seo-gu, Busan 49267, Republic of Korea; kimhs.onco@gmail.com (H.S.K.); jwshim@kosin.ac.kr (J.S.); 2 Department of Gastroenterology, Ajou University School of Medicine, 164 Worldcup-ro, Yeongtong-gu, Suwon 16499, Republic of Korea; csh3601@naver.com (S.H.J.); ptok99@hanmail.net (G.O.B.); ymk8028@hanmail.net (M.G.Y.); overdream@nate.com (J.E.H.); cocorico99@gmail.com (S.S.K.); jaeyoun620@gmail.com (J.Y.C.); 3 Department of Biomedical Sciences, Ajou University Graduate School of Medicine, 164 Worldcup-ro, Yeongtong-gu, Suwon 16499, Republic of Korea

**Keywords:** Hepatocellular carcinoma, long non-coding RNA, *CTC-537E7.3*, diagnostic biomarker, competing endogenous RNA

## Abstract

Hepatocellular carcinoma (HCC) critically lacks reliable biomarkers for early detection. By mining the TCGA_LIHC and two GEO cohorts, we identified the liver-specific long non-coding RNA *CTC-537E7.3* as the most consistently down-regulated transcript in tumors. This finding was validated in 97 paired tissues, with *CTC-537E7.3* expression lost in 95% of cases (*** *p* < 0.0001). It demonstrated excellent diagnostic performance in discriminating tumor from non-tumor tissue (AUC = 0.95), which was maintained in early-stage (I/II) disease. Low *CTC-537E7.3* expression correlated with shorter overall and disease-free survival and was inversely associated with serum α-fetoprotein (AFP) levels, highlighting its complementary clinical value. Mechanistic investigation revealed a potential competing endogenous RNA (ceRNA) axis. The microRNA miR-190b-5p was highly expressed in tumors and predicted to bind *CTC-537E7.3*, while its target, *PLGLB1*, was significantly suppressed. Survival analysis confirmed that concurrent high expression of *CTC-537E7.3* and *PLGLB1* conferred superior outcomes. These findings establish *CTC-537E7.3* as a liver-specific, ceRNA-mediated tumor suppressor with robust diagnostic and prognostic potential. It represents a promising adjunct to existing HCC surveillance strategies, such as ultrasound and AFP measurement, for high-risk populations.

## 1. Introduction

Hepatocellular carcinoma (HCC), the sixth most frequently diagnosed type of cancer, is the third leading cause of cancer-related mortality worldwide, with approximately 900,000 new cases and 830,000 deaths [1,2]. The risk factors for HCC include chronic hepatitis B or C infection, alcoholic liver disease, and nonalcoholic fatty liver disease (NAFLD) [3]. Owing to the asymptomatic nature of the early stages, most patients present with advanced stages of HCC at the time of diagnosis despite the advancements in surveillance programs. This contributes to a poor 5-year survival rate of <20% [4].

The early and accurate detection of HCC remains a pivotal challenge in clinical practice. Alpha-fetoprotein (AFP) and proteins induced by vitamin K absence II (PIVKA-II) are the primary serum biomarkers used for HCC surveillance at present; however, the sensitivity (62–65%) and specificity (approximately 87%) of AFP are often insufficient for the detection of early-stage HCC. Furthermore, AFP levels may be elevated in patients with non-malignant liver conditions [5]. The sensitivity and specificity of PIVKA-II are approximately 71% and 90% [5], respectively; nevertheless, it may fail to detect a subset of tumors. Moreover, the lens culinaris agglutinin-reactive fraction of AFP (AFP-L3) has emerged as a guideline-endorsed refinement that increases HCC specificity when used alongside total AFP and PIVKA-II, yet its stand-alone sensitivity—especially for early tumors—remains modest and its global adoption uneven [6,7]. The limitations of these biomarkers underscore the necessity of developing novel diagnostic tools for the early detection and risk stratification of HCC.

Long non-coding RNAs (lncRNAs), defined as RNA transcripts exceeding 200 nucleotides without protein-coding potential, are promising candidates that could serve as cancer biomarkers [8,9]. The roles of lncRNAs encompass a broad spectrum of regulatory functions, including epigenetic remodeling, modulation of transcription factor activity, and microRNA sequestration [10,11,12,13]. Beyond their functional versatility, lncRNAs possess the following intrinsic properties that favor clinical translation: (i) tightly regulated, tissue- and cell-type-specific transcription driven by epigenetic programs; (ii) complex secondary/tertiary structures that confer resistance to ribonuclease degradation; and (iii) selective packaging into extracellular vesicles, enabling stable and non-invasive detection in biofluids such as serum, plasma, and urine [8,14,15,16]. These advantages are already reflected in the clinic; for example, the prostate-specific lncRNA PCA3 received FDA approval in 2012 for urine-based testing [17] and, in bladder cancer, *UCA1* has achieved >90% sensitivity and >95% specificity in prospective studies and is currently undergoing multicenter validation [18]. Furthermore, a multicenter study showed that serum extracellular-vesicle-derived LINC00853 discriminated all-stage HCC from non-HCC liver disease with an AUC of 0.93 (95% CI, 0.887–0.966) and outperformed AFP in early (mUICC stage I) tumors, achieving 94% sensitivity and 90% specificity versus 9% and 73% for AFP; notably, LINC00853 remained positive in 97% of AFP-negative early HCC cases [19]. The specificity and functional relevance of lncRNA-based biomarkers have prompted extensive research on these biomarkers to improve the diagnosis and prognosis of multiple types of cancer.

The present study focuses on a novel liver-specific lncRNA, *CTC-537E7.3*, which was significantly downregulated in HCC across multiple transcriptomic datasets. The diagnostic and prognostic potential of *CTC-537E7.3* was further validated through quantitative real-time polymerase chain reaction (qRT-PCR) analysis of 97 paired HCC and adjacent non-tumor (NT) tissues that examined its correlations with clinicopathological parameters and patient outcomes. Moreover, a dedicated competitive endogenous RNAs (ceRNAs) analysis suggested that *CTC-537E7.3* may act as a molecular sponge for the tumor-suppressive miR-190b-5p, thereby relieving post-transcriptional repression of the oncogenic effector *PLGLB1*. The present study reveals that *CTC-537E7.3* is a clinically relevant biomarker with high specificity for liver tissue that could serve as a potential adjunct to AFP in facilitating the early detection and risk stratification of HCC.

## 2. Materials and Methods

### 2.1. Expression and Prognostic Analysis in Public Omics Datasets

Transcriptomic data obtained from three independent datasets, The Cancer Genome Atlas-Liver Hepatocellular Carcinoma (TCGA_LIHC), Gene Expression Omnibus (GEO) series GSE77314 [20], and series GSE124535 [21], are used in the present study. The lncRNAs extracted from these datasets were filtered through differential expression analysis (*** *p* < 0.001) of the NT and tumor (T) samples. Prior to this test, a biologically driven expression filter was applied—only lncRNAs with log_2_(TPM + 1) > 1 (≈TPM > 1) in ≥70% of NT samples and log_2_(TPM + 1) < 1 in ≥70% of T samples were retained. TPM ≈ 1 is widely accepted as the lower bound for reliable detection and corresponds to roughly one transcript per cell; using this threshold enriches for genes that are robustly expressed in normal liver yet effectively silenced in HCC, thereby minimizing artefactual fold changes from transcripts with uniformly low counts. The 10 most downregulated lncRNAs across all datasets were identified for further analyses.

The TCGA_LIHC dataset was used to further validate the expression levels in the NT vs. T samples. Kaplan–Meier survival analysis was performed to evaluate the prognostic relevance. The duration between the diagnosis of HCC and death from any cause was defined as overall survival (OS). The duration between curative treatment and recurrence of disease was defined as disease-free survival (DFS). The duration between the diagnosis of HCC and death caused by HCC, excluding deaths from other causes, was defined as disease-specific survival (DSS). The duration between the diagnosis of HCC and disease progression or recurrence was defined as progression-free survival (PFS). Two complementary analyses were conducted to determine the liver-specific expression of *CTC-537E7.3*. Transcriptomic data from the Genotype-Tissue Expression (GTEx) project (https://www.gtexportal.org/home/, accessed on 9 July 2025) were retrieved to compare the expression across a wide range of normal tissues. Subsequently, pan-cancer expression data across 23 different types of cancer were obtained from the Genomic Data Commons (GDC) hub of UCSC Xena (https://xena.ucsc.edu/, accessed on 9 July 2025) to examine the levels in the T and NT tissues [22].

### 2.2. qRT-PCR

QIAzol Reagent (Qiagen, Hilden, Germany) was used in accordance with the manufacturer’s instructions to extract total RNA from the frozen tissue samples. Subsequently, 5× PrimeScript™ RT Master Mix (Takara Bio, Shiga, Japan) was used to synthesize cDNA from 500 ng of total RNA under the following conditions: 37 °C for 15 min, 85 °C for 5 s, and 4 °C. AmfiSure qGreen Q-PCR Master Mix (GenDEPOT, Barker, TX, USA) was used to conduct qRT-PCR on a CFX Connect Real-Time PCR Detection System (Bio-Rad Laboratories, Hercules, CA, USA). The expression levels were normalized to those of HMBS as internal control. The amplification *CTC-537E7.3* primer sequences were 5′-GGG AGG AAC CCG TTT GGA TT-3′ (forward) and 5′-GAC CTT CCC AGG ACC TCA AC-3′ (reverse). The *HMBS* primer sequences were 5′-ACGGCTCAGATAGCATACAAGAG-3′ (forward) and 5′-GTTACGAGCAGTGATGCCTACC-3′ (reverse). PCR was conducted under the following conditions: 95 °C for 2 min; 40 cycles at 95 °C for 15 s, 62 °C for 34 s, and 72 °C for 30 s; followed by a dissociation stage at 95 °C for 10 s, 65 °C for 5 s, and 95 °C for 5 s. The relative expression was determined using the relative standard curve method (2^−ΔΔCt^). All experiments were performed at least in triplicate.

### 2.3. Clinical Sample Collection

Ninety-seven paired HCC and corresponding NT liver tissue samples were obtained from the Biobank of Ajou University Hospital (Suwon, Republic of Korea) to validate the expression patterns of *CTC-537E7.3* observed from public omics data. qRT-PCR analysis was conducted as described previously. Comprehensive demographic and clinical data, including age, sex, etiology of liver disease, body mass index (BMI), platelet count, serum albumin levels, total bilirubin levels, international normalized ratio (INR), creatinine levels, sodium levels, aspartate aminotransferase (AST) levels, alanine aminotransferase (ALT) levels, AFP levels, PIVKA-II levels, hemoglobin levels, glucose levels, total cholesterol levels, and the presence of ascites, were recorded. Tumor staging was performed in accordance with the Pathological Union for International Cancer Control (pUICC) classification system (Table 1).

### 2.4. miRNA Expression Profiling and ceRNA Network Construction

Raw mature-miRNA read counts for TCGA_LIHC were downloaded from the GDC using the TCGAbiolinks pipeline (v2.30.1) [23,24]. Counts were filtered to retain miRNAs expressed at ≥1 count per million (CPM) in ≥30% of samples, then TMM-normalized and transformed to log_2_-CPM with the edgeR package (v4.2). Differential expression between non-tumor (NT) and tumor (T) tissues were assessed with limma-voom (v3.58); miRNAs with |log_2_ FC| ≥ 0.5 and FDR < 0.05 were considered significantly deregulated.

For ceRNA screening, Pearson correlation coefficients (r) were calculated between *CTC-537E7.3* and each miRNA across matched mRNA- and miRNA-seq tumor samples. Candidate oncogenic miRNAs were defined as follows: (i) overexpressed in tumors and (ii) inversely correlated with *CTC-537E7.3*. Putative mRNA targets of each candidate miRNA were retrieved from miRDB (https://mirdb.org/, accessed on 9 July 2025) with a target score ≥ 70 [25]. Predicted targets were filtered to keep transcripts that were as follows: (i) downregulated in tumors and (ii) positively correlated with *CTC-537E7.3* while showing a negative correlation with the cognate miRNA.

### 2.5. Statistical Analysis

All data are presented as mean ± standard deviation (SD). Inter-group differences were assessed using an unpaired Student’s *t*-test (GraphPad Software, version 10.0; San Diego, CA, USA). Kaplan–Meier survival curves were generated for OS, DFS, DSS, and PFS. Statistical significance was determined using the log-rank test. MedCalc (MedCalc Software, version 22.018; Ostend, Belgium), which provided AUC, 95% confidence interval (CIs), sensitivity, and specificity, was used to conduct receiver operating characteristic (ROC) curve analyses. Statistical significance was set at * *p* < 0.05. All experiments were performed at least in triplicate.

## 3. Results

### 3.1. Identification of CTC-537E7.3 as a Potential Diagnostic Biomarker for Liver Cancer

**Figure 1:**
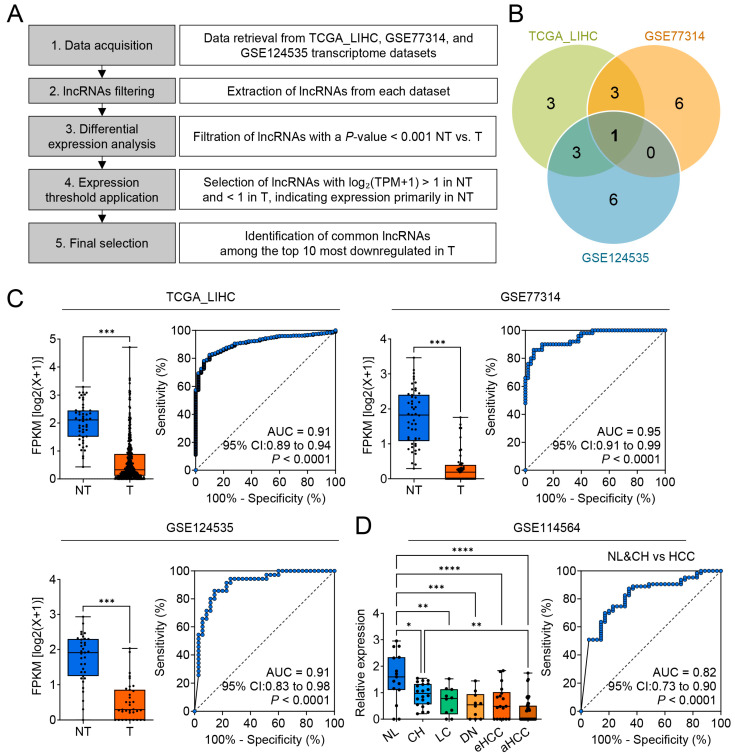
Integrative Analysis of Differentially Expressed lncRNAs in HCC Across Multiple Datasets. (**A**) Flow diagram detailing the methodology used to identify differentially expressed lncRNAs in HCC. (**B**) Venn diagram illustrating the overlap of significantly differentially expressed lncRNAs across the TCGA_LIHC, GSE77314, and GSE124535 datasets. (**C**) Box plots (left bar plot of the each dataset) showing the expression levels of *CTC-537E7.3* in tumor (T) and non-tumor (NT) liver tissues across TCGA_LIHC, GSE77314, and GSE124535 datasets, demonstrating its significantly downregulation in HCC compared to NT. Receiver operating characteristic (ROC) curves (right ROC curve plot of the each dataset) demonstrate the diagnostic performance of *CTC-537E7.3* in distinguishing HCC from non-tumor liver tissues, presenting area under the curve (AUC) values, 95% confidence intervals (CIs), and statistical significance (*p*-values). (**D**) Expression levels of *CTC-537E7.3* across different stages of liver disease and cancer progression, including normal liver (NL), chronic hepatitis (CH), liver cirrhosis (LC), dysplastic nodules (DN), early HCC (eHCC), and advanced HCC (aHCC), based on the GSE114564 dataset (left panel). The Y-axis represents the expression levels of *CTC-537E7.3*, while the X-axis denotes different sample groups. Statistical significance between groups is indicated by asterisks (* *p* < 0.05, ** *p* < 0.01, *** *p* < 0.001, **** *p* < 0.0001).

Transcriptomic data from TCGA-LIHC and two GEO cohorts (GSE77314, GSE124535) were analyzed with a two-stage filter. First, lncRNAs significantly downregulated in tumors versus matched normal tissue (*p* < 0.001) were selected. Second, candidates had to show log_2_(TPM + 1) > 1 in normal tissue but <1 in tumors, enriching for transcripts robustly expressed in healthy liver yet effectively silenced in HCC (Figure 1A). Among the ten most down-regulated lncRNAs in each dataset, only *CTC-537E7.3* was common to all three (Figure 1B; Table 2), underscoring its consistent loss during hepatocarcinogenesis. Consistent with this two-stage selection, we next validated *CTC-537E7.3* expression across all three cohorts. *CTC-537E7.3* expression in the T tissues was significantly lower than that in the NT tissues across the three datasets (TCGA_LIHC, GSE77314, and GSE124535) (Figure 1C, left bar plots for each dataset). Notably, the expression level was maintained above the threshold (fragments per kilobase per million [FPKM] log_2_ > 1) in the NT tissues but below the threshold (FPKM log_2_ < 1) in the T tissues, underscoring tumor-specific downregulation. This pattern of further reduction from a relatively low basal level suggests that *CTC-537E7.3* may play a tumor-suppressive role.

ROC curve analysis conducted to evaluate the diagnostic potential of *CTC-537E7.3* revealed that *CTC-537E7.3* exhibited high discriminatory power in distinguishing tumor tissues from normal tissues, yielding AUC values of 0.91 (95% CI: 0.89–0.94, *** *p* < 0.0001), 0.95 (95% CI: 0.91–0.99, *** *p* < 0.0001), and 0.91 (95% CI: 0.83–0.98, *** *p* < 0.0001) in TCGA_LIHC, GSE77314, and GSE124535, respectively (Figure 1C, right ROC curve plots for each dataset). The association between *CTC-537E7.3* expression and the progression of liver disease in different stages of liver disease, including normal liver (NL), chronic hepatitis (CH), liver cirrhosis (LC), dysplastic nodules (DN), early HCC (eHCC), and advanced HCC (aHCC), was further assessed using the GSE114564 dataset (Figure 1D, left bar plot). This analysis revealed that *CTC-537E7.3* expression was significantly reduced as disease severity increased. However, *CTC-537E7.3* expression in CH did not differ significantly from that in NL. These findings indicate that its downregulation was more specifically linked to malignant transformation.

*CTC-537E7.3* could effectively distinguish noncancerous liver diseases (NL and CH) from HCC, achieving an AUC of 0.82 (95% CI: 0.73–0.90, *** *p* < 0.0001) in the ROC analysis (Figure 1D, right). Additionally, to test whether pre-existing cirrhosis attenuates this diagnostic performance, we analyzed an independent cohort (GSE104310) in which NT and HCC specimens are explicitly stratified by cirrhosis status. *CTC-537E7.3* remained significantly lower in HCC than in NT both for non-cirrhotic livers (AUC = 0.90, *** *p* = 0.003) and cirrhotic livers (AUC = 1.00, * *p* = 0.02) (Supplementary Figure S1A,B).

These findings suggest its potential utility in the early detection and precise disease stratification of HCC, further suggesting that *CTC-537E7.3* may act as a valuable biomarker for monitoring the progression of benign or precancerous liver conditions to overt malignancy.

### 3.2. Prognostic Relevance of CTC-537E7.3 in Patients with HCC

**Figure 2:**
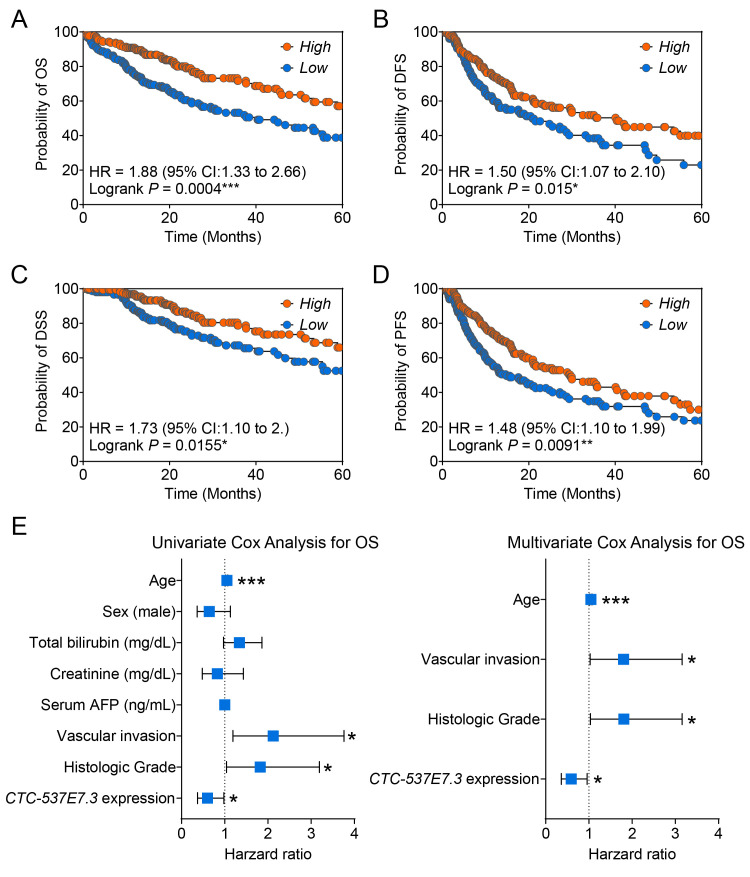
Kaplan–Meier survival analyses illustrating the prognostic significance of *CTC-537E7.3* expression in HCC patients based on the TCGA_LIHC dataset. (**A**) Overall survival (OS), (**B**) disease-free survival (DFS), (**C**) disease-specific survival (DSS), and (**D**) progression-free survival (PFS) analyses. Patients were stratified into high- and low-expression groups based on median *CTC-537E7.3* expression levels (orange: high expression, blue: low expression). (**E**) Forest plot summarizing the HRs and 95% CIs obtained from univariate and multivariate Cox proportional-hazards models for overall survival in the TCGA-LIHC cohort. Hazard ratios (HRs), 95% confidence intervals (CIs), and log-rank *p*-values are presented. Statistically significant differences were determined using the log-rank test (* *p* < 0.05, ** *p* < 0.01, *** *p* < 0.001).

Kaplan–Meier survival analyses were conducted using the TCGA_LIHC cohort to evaluate the correlation between *CTC-537E7.3* expression and patient outcomes. The median expression levels were used to stratify the patients into high- and low-expression groups. Notably, low *CTC-537E7.3* expression exhibited a significant association with poor outcomes across multiple endpoints. A marked reduction in the OS was observed in the low-expression group (hazard ratio [HR] = 1.88, *p* = 0.0004; Figure 2A), indicating a higher risk of death among patients with diminished *CTC-537E7.3* levels. The DFS in this group was also shorter (HR = 1.50, * *p* = 0.015; Figure 2B), indicating an increased likelihood of early recurrence. Furthermore, low *CTC-537E7.3* expression was also associated with poorer DSS (HR = 1.73, * *p* = 0.0155; Figure 2C) and PFS (HR = 1.48, ** *p* = 0.0091; Figure 2D), underscoring its potential as a broad indicator of tumor aggressiveness.

To determine whether the prognostic effect of *CTC-537E7.3* is independent of major clinicopathological factors, we next performed comprehensive Cox regression modeling in the TCGA-LIHC cohort (n = 188). Univariate analysis confirmed that low *CTC-537E7.3* expression was associated with shorter OS, DFS, and PFS. Similar trends were observed for DFS (adjusted HR = 0.76, *p* = 0.082) and PFS (adjusted HR = 0.75, *p* = 0.062), whereas an effect on DSS did not reach statistical significance (Supplementary Table S1). Importantly, after adjustment for vascular invasion, histologic grade, age, and other covariates, *CTC-537E7.3* remained an independent protective factor for OS (multivariate HR = 0.59, 95% CI 0.36–0.96, * *p* = 0.033) (Figure 2E).

Although propensity-score matching was considered, including multiple clinical variables markedly reduced the evaluable sample size and statistical power; the full-cohort multivariate approach thus provides a more robust and clinically representative assessment. Collectively, these analyses reinforce *CTC-537E7.3* as an independent, favorable prognostic biomarker in HCC, even after accounting for established risk factors.

### 3.3. Tissue Specificity of CTC-537E7.3 in Liver and Cancer Contexts

**Figure 3:**
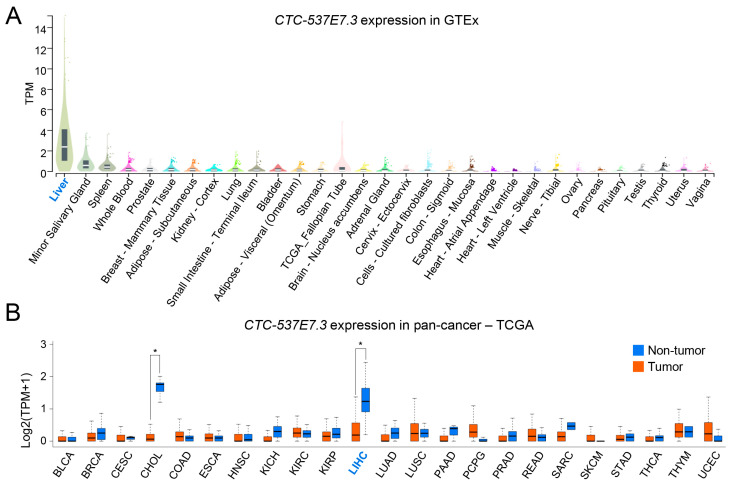
*CTC-537E7.3* Expression in Normal and Cancerous Tissues. (**A**) *CTC-537E7.3* expression across various normal human tissues from the GTEx dataset. The Y-axis represents log_2_(TPM + 1). (**B**) Pan-cancer analysis of *CTC-537E7.3* expression across 23 cancer types using TCGA data. Significant differences in expression between tumor (tumor, red box) and normal adjacent tissues (non-tumor, blue box) are highlighted for hepatocellular carcinoma (LIHC) and cholangiocarcinoma (CHOL). BLCA, bladder urothelial carcinoma; BRCA, breast invasive carcinoma; CESC, cervical squamous cell carcinoma and endocervical adenocarcinoma; COAD, colon adenocarcinoma; ESCA, esophageal carcinoma; HNSC, head and neck squamous cell carcinoma; KICH, kidney chromophobe; KIRC, kidney renal clear cell carcinoma; KIRP, kidney renal papillary cell carcinoma; LUAD, lung adenocarcinoma; LUSC, lung squamous cell carcinoma; PAAD, pancreatic adenocarcinoma; PCPG, pheochromocytoma and paraganglioma; PRAD, prostate adenocarcinoma; READ, rectum adenocarcinoma; SARC, sarcoma; SKCM, skin cutaneous melanoma; STAD, stomach adenocarcinoma; THCA, thyroid carcinoma; THYM, thymoma; UCEC, uterine corpus endometrial carcinoma. * *p* < 0.05.

Several lncRNAs, even those with relatively low basal expression, exhibit distinct tissue-specific patterns [26]. Therefore, data from the GTEx project were analyzed to determine whether CTC-537E7.3 exhibits a similar trend. Notably, the *CTC-537E7.3* expression in the liver was elevated; in contrast, its expression was minimal or undetectable in other organs (Figure 3A). This pronounced specificity indicates that *CTC-537E7.3* is predominantly associated with the liver under physiological conditions. A pan-cancer analysis was conducted using TCGA data from 23 types of cancer. Consistent with the GTEx findings, *CTC-537E7.3* expression in LIHC and cholangiocarcinoma (CHOL) was significantly downregulated compared with that in their respective NT counterparts (Figure 3B). Notably, *CTC-537E7.3* expression was negligible in the T and adjacent NT tissues in other types of malignancies. Collectively, these results underscore the strong liver specificity of *CTC-537E7.3*, emphasizing its potential value as a biomarker for the detection of liver cancer, given the marked difference in its expression in LIHC and CHOL compared with that in the NT liver tissues.

### 3.4. Validation of CTC-537E7.3 as a Diagnostic Biomarker in Patients with HCC

**Figure 4:**
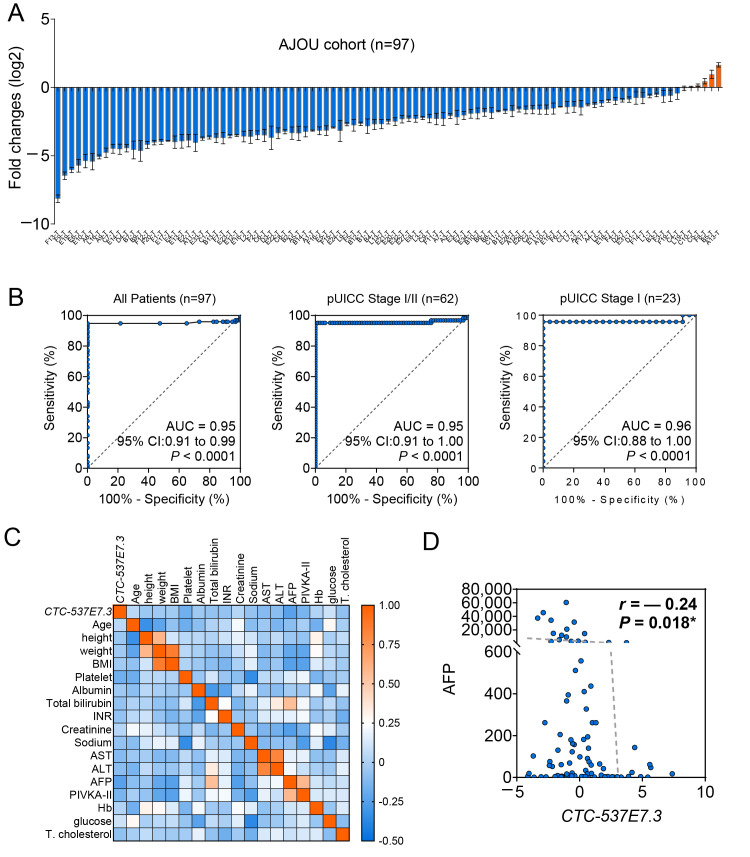
*CTC-537E7.3* Expression and Diagnostic Potential in HCC Patients. (**A**) qRT-PCR analysis of *CTC-537E7.3* expression in paired tumor (T) and adjacent non-tumor (NT) tissues from 97 HCC patients. The X-axis represents each patient’s unique ID and the X-axis indicates the log_2_ fold change. (**B**) Receiver operating characteristic (ROC) curves assessing the diagnostic performance of *CTC-537E7.3* in distinguishing HCC from non-tumor tissues across all patients (n = 97), early-stage HCC patients (pUICC I/II, n = 62), and stage I HCC patients (pUICC I, n = 23). Area under the curve (AUC) values, 95% confidence intervals (CIs), and *P*-values are provided. (**C**) Correlation analysis between *CTC-537E7.3* expression and clinical parameters, including AFP levels. (**D**) Spearman correlation analysis between *CTC-537E7.3* expression and serum AFP levels in HCC patients (n = 97). The correlation coefficient (r) and *p*-value are indicated. Statistical significance was defined as * *p* < 0.05.

Given the pronounced tissue specificity and consistent downregulation in HCC, the diagnostic utility of *CTC-537E7.3* was evaluated in a clinical cohort. qRT-PCR analysis was conducted using 97 matched pairs of T and NT liver tissue samples. A significant reduction in *CTC-537E7.3* expression was observed in 91 of the 97 cases (95%) (*** *p* <0.0001), confirming its recurrent loss in T tissues (Figure 4A). ROC curve analysis conducted to further assess the diagnostic performance of *CTC-537E7.3* demonstrated its strong ability to distinguish HCC from NT tissues, yielding an AUC of 0.95 (95% CI: 0.91–0.99, *** *p* < 0.0001). Notably, *CTC-537E7.3* retained this diagnostic power even for the detection of early-stage HCC, achieving AUCs of 0.95 (95% CI: 0.91–1.00, *** *p* < 0.0001) and 0.96 (95% CI: 0.88–1.00, *** *p* < 0.0001) in patients with pUICC stage I/II disease (n = 62) and those with pUICC stage I disease (n = 23), respectively (Figure 4B). These findings are consistent with those of previous ROC analyses conducted using the TCGA_LIHC, GSE77314, and GSE124535 datasets, reinforcing the robustness of *CTC-537E7.3* as a diagnostic biomarker across independent cohorts. A correlation heatmap analysis was conducted to examine the association between *CTC-537E7.3* expression and various clinical parameters to determine its clinical relevance (Figure 4C). The heatmap revealed a general trend of negative correlation, with the darker blue shading indicating a stronger negative association. Notably, *CTC-537E7.3* expression exhibited a statistically significant correlation with serum AFP levels only (*r* = −0.24, * *p* = 0.018). The levels of AFP, a widely used biomarker for HCC, increase in response to liver damage and are particularly elevated in patients with HCC. However, given the limitations of AFP as a stand-alone diagnostic marker, the findings of the present study suggest that *CTC-537E7.3* may serve as a complementary biomarker to enhance the accuracy of the diagnosis of HCC and improve patient stratification (Figure 4D). The combination of *CTC-537E7.3* and AFP may represent an improved strategy for the detection of early-stage HCC and more precise disease classification.

### 3.5. CTC-537E7.3 Acts as a ceRNA That Relieves miR-190b-5p-Mediated Repression of PLGLB1 in HCC

**Figure 5:**
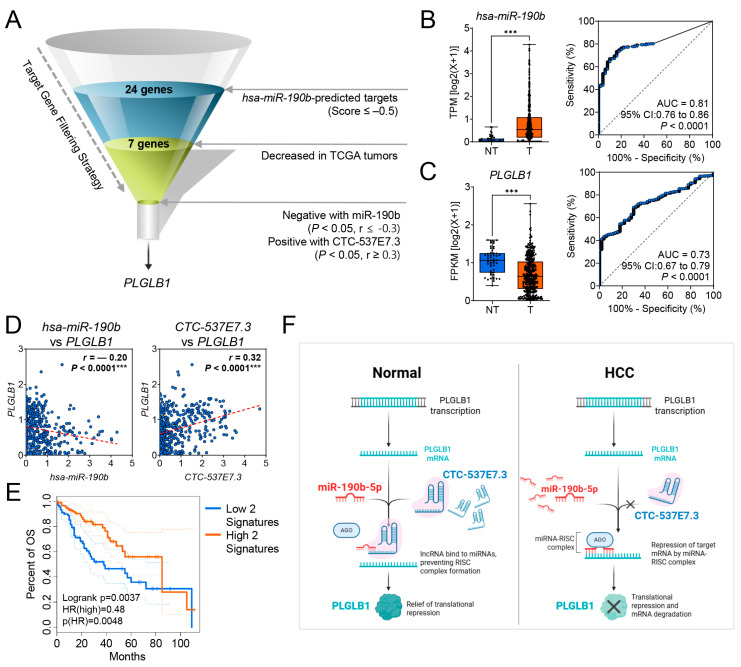
The *CTC-537E7.3*–miR-190b-5p*–PLGLB1* ceRNA Axis in HCC. (**A**) Funnel diagram illustrating the step-wise screening strategy used to identify key components of the ceRNA axis. The process filtered miR-190b-5p targets from miRDB, selected for genes with tumor-specific downregulation, and identified *PLGLB1* as the single gene showing an inverse correlation with miR-190b-5p and a positive correlation with *CTC-537E7.3* in the TCGA_LIHC dataset. (**B**) Box plot (left) comparing miR-190b-5p expression in non-tumor (NT) and tumor (T) liver tissues, and a receiver operating characteristic (ROC) curve (right) demonstrating its diagnostic performance. (**C**) Box plot (left) showing *PLGLB1* expression in NT versus T tissues, with a corresponding ROC curve (right) illustrating its diagnostic accuracy. For both (**B**,**C**), area under the curve (AUC), 95% confidence intervals (CIs), and *p*-values are provided. (**D**) Pearson correlation analysis showing the inverse relationship between miR-190b-5p and *PLGLB1* (left), and the positive relationship between *CTC-537E7.3* and *PLGLB1* (right). Correlation coefficients (r) and *p*-values are indicated. (**E**) Kaplan–Meier overall survival analysis based on combined expression signatures. Patients were stratified into a concordant low-expression group (concurrent low expression of both *CTC-537E7.3* and *PLGLB1*) and a concordant high-expression group (concurrent high expression of both transcripts). Hazard ratio (HR), 95% CI, and the log-rank *p*-value are presented. (**F**) Schematic model of the proposed mechanism. In normal hepatocytes (left), *CTC-537E7.3* sponges miR-190b-5p, allowing *PLGLB1* translation. In HCC (right), reduced *CTC-537E7.3* levels lead to increased miR-190b-5p activity, resulting in the repression and degradation of *PLGLB1*. Statistical significance between groups is indicated by asterisks (*** *p* < 0.001).

lncRNAs are well known to regulate gene expression by sequestering miRNAs within ceRNA networks [27]. To select the most biologically relevant miRNA interactor for *CTC-537E7.3*, we first extracted the top 10 miRNAs with the highest target scores from miRDB and cross-referenced their expression in the TCGA-LIHC cohort using the ENCORI Pan-Cancer platform (Figure 5A; Supplementary Table S2). Among these candidates, only miR-190b-5p was significantly over-expressed in tumor versus normal liver (fold change = 8.36, FDR = 1.4 × 10^−9^), thereby meeting both in silico affinity and in vivo dysregulation criteria.

Having established miR-190b-5p as the prime candidate, we next evaluated its downstream network components. In HCC, miR-190b-5p was the most abundantly expressed miRNA predicted to bind *CTC-537E7.3* (*** *p* < 0.0001; Figure 5B, left), whereas its top anti-correlated mRNA target, *PLGLB1*, was markedly down-regulated (*** *p* < 0.0001; Figure 5C, left; Supplementary Table S3). ROC curves showed good diagnostic accuracy for both miR-190b-5p (AUC ≈ 0.81) and *PLGLB1* (AUC ≈ 0.73) (Figure 5B,C, right panels). Pair-wise Pearson correlation analysis revealed a strong inverse correlation between miR-190b-5p and *PLGLB1* (r = −0.20, *** *p* < 0.0001) and a positive correlation between *CTC-537E7.3* and *PLGLB1* (r = 0.32, *** *p* < 0.0001; Figure 5D). Collectively, these observations reinforce the notion that *CTC-537E7.3* and *PLGLB1* constitute a miR-190b-5p–mediated ceRNA pair that reciprocally modulates each other’s expression. Kaplan–Meier analysis stratified patients into a Low-2 signature (concurrent low expression of both *CTC-537E7.3* and *PLGLB1*) and a High-2 signature (concurrent high expression of both transcripts). The High-2 group experienced significantly better overall survival than the Low-2 group (HR = 0.48, 95% CI 0.29–0.80; log-rank *** *p* = 0.0048; Figure 5E; Supplementary Figure S2), underscoring their combined prognostic value.

Collectively, these observations support a mechanistic model in which abundant *CTC-537E7.3* in normal hepatocytes binds miR-190b-5p, preventing RISC assembly on *PLGLB1* transcripts and permitting translation. In HCC, reduced *CTC-537E7.3* releases miR-190b-5p, enhancing RISC-mediated repression and degradation of *PLGLB1* (Figure 5F).

## 4. Discussion

lncRNAs, often exceeding 200 nucleotides and lacking coding capacity, have emerged as pivotal regulators in the field of oncology, reflecting a consensus shift that recognizes these transcripts as active participants in gene regulation rather than merely transcriptional noise [8,28]. The majority of lncRNAs comprise intricate secondary and tertiary structures that enable the formation of ribonucleoprotein complexes that modulate transcription, translation, and epigenetic landscapes [29].

Several studies have identified multiple dysregulated lncRNAs with oncogenic or tumor-suppressive functions that contribute to tumor progression in patients with liver cancer. TUC339, a liver-specific lncRNA notable for its enrichment in extracellular vesicles (EVs), has demonstrated the selective packaging and secretion of lncRNAs to influence macrophage polarization and distant metastatic niches [30]. These findings underscore the broad potential of lncRNAs to serve as diagnostic and prognostic biomarkers given their tissue specificity and stability in biofluids [31]. Although AFP often fails to detect early-stage HCC [32], EV lncRNAs outperform or complement traditional markers.

The present study focuses on *CTC-537E7.3*, an intergenic lncRNA located on chromosome 5q13.1 that has received little attention prior to high-throughput analyses indicating its aberrant expression in HCC. The present study demonstrates the downregulation of *CTC-537E7.3* in T tissue samples across independent cohorts and its strong correlation with adverse clinicopathological features. Furthermore, the present study demonstrates that *CTC-537E7.3* may serve as an effective biomarker for distinguishing HCC from NT liver tissue. In our biopsy cohort, qRT-PCR quantification of *CTC-537E7.3* achieved an AUC > 0.95—even in stage I disease—surpassing the routinely used histological markers HepPar-1, arginase-1, and glypican-3 [33,34]. Importantly, the transcript’s performance remained robust when combined with these classical markers, suggesting that *CTC-537E7.3* can complement rather than replace histological assessment while providing fully quantitative, highly reproducible read-outs.

Further clinical evaluation revealed associations with poorer patient outcomes, indicating that *CTC-537E7.3* could distinguish individuals at a higher risk of disease progression, thereby serving as a prognostic marker. These findings established patterns observed in other tumor-suppressive lncRNAs, underscoring that *CTC-537E7.3* may help regulate key oncogenic pathways in the liver. Additionally, our ceRNA analysis indicates that *CTC-537E7.3* acts as a sponge for the oncogenic miR-190b-5p, thereby leading to repression of *PLGLB1*; perturbation of this axis may enhance *PLGLB1* expression and drive tumor progression.

Advances in the field of lncRNA-based diagnostics, particularly for HCC, have emphasized the feasibility of incorporating new biomarkers alongside existing standards, such as AFP. For instance, lncRNA assays can facilitate early detection of HCC in at-risk populations or refine risk stratification after curative treatment [35,36]. Establishing standardized protocols for measuring *CTC-537E7.3* in routine practice may facilitate the integration of lncRNA profiles into current surveillance strategies.

From a translational standpoint, the tumor-specific downregulation and predictive capacity of *CTC-537E7.3* could potentially aid in disease monitoring and stratification of patients for surveillance, especially if future work identifies a robust detection platform for clinical application. Collectively, the findings of the present study indicate that *CTC-537E7.3* may serve as a potentially meaningful biomarker for HCC and underscore the broader concept that lncRNAs, by virtue of their tissue specificity and regulatory capacity, may elucidate tumor biology and enhance diagnostic strategies.

## 5. Conclusions

In summary, the present study reveals that *CTC-537E7.3* is a promising liver-specific lncRNA biomarker that may aid in the early detection and prognostication of HCC. The downregulation of *CTC-537E7.3* in tumor tissues, coupled with its strong discriminatory power in differentiating HCC from NT liver samples, underscores its diagnostic utility. Our results also revealed a putative *CTC-537E7.3*/miR-190b-5p/PLGLB1 ceRNA circuit, which may contribute to tumor progression in HCC. The significant association observed between low *CTC-537E7.3* expression and poor survival outcomes highlights its prognostic value. Future studies must be conducted in the future to clarify the underlying mechanisms and validate the clinical applicability of *CTC-537E7.3*. Nevertheless, *CTC-537E7.3* broadens the spectrum of lncRNA-based approaches, offering a tangible step toward more precise management of HCC.

## Figures and Tables

**Table 1:** Clinicopathological characteristic of the patients in Ajou hospital cohort.

Variables	HCC (n = 97)
Age (years), mean ± SD	55.7 ± 10.0
Male sex, n (%)	74 (76.3%)
Etiology, n (%)	
HBV	90 (92.8%)
HCV	4 (4.1%)
Alcohol	2 (2.1%)
HCV + Alcohol	1 (1%)
Cirrhosis, n (%)	54 (65.1)
Ascites, n (%)	16 (16.3)
BMI	24.2 (387)
Platelet, ×10^9^/L	179.1 (70.1)
Albumin (g/dL), mean ± SD	4.4 (0.7)
Total bilirubin (mg/dL), mean ± SD	0.8 (0.9)
INR, mean ± SD	1.1 (0.1)
Creatinine (mg/dL)	0.9 (0.3)
Sodium, mmol/L	115.7 (52.3)
AST, U/L	54.3 (105.3)
ALT, U/L	42.5 (61.1)
AFP (ng/mL), mean ± SD	94.9 (144.8)
PIVKA-II, mAU/mL	8925.3 (19,566.8)
Hemoglobin, g/dL	14.4 (1.6)
Glucose, mg/dL	122.3 (53.6)
Total Cholesterol, mg/dL	173.7 (40.4)
pUICC stage, n (%)	
pI	24 (24.7)
pII	38 (39.2)
pIII	24 (24.7)
pIVA	7 (7.2)
pIVB	3 (3.1)
BCLC stage, n (%)	
0	24 (24.7)
A	53 (54.6)
B	8 (8.2)
C	9 (9.3)
D	2 (2.1)
Vascular invasion, n (%)	47 (56)
Metastasis, n (%)	22 (22.7)
Recurrence, n (%)	38 (39.2)
OS (months), mean ± SD	46.0 (24.5)

HCC: hepatocellular carcinoma, SD: standard deviation, HBV: hepatitis B virus, HCV: hepatitis C virus, BMI: body mass index, INR: international normalized ratio, AST: aspartate aminotransferase, ALT: alanine aminotransferase, AFP: alpha-fetoprotein, PIVKA-II: protein induced by vitamin K absence-II, pUICC: pathological union for international cancer control, BCLC: Barcelona clinic liver cancer, OS: overall survival, mAU/mL: milli-arbitrary units per milliliter.

**Table 2:** The expression of top 10 lncRNAs in HCC Across TCGA and GEO Cohorts.

Dataset	Rank	Gene Symbol	Gene ID	*p*-Value	Fold Change (log_2_)	Normal Expression	Tumor Expression
TCGA_LIHC	1	*RP11-6B4.1*	ENSG00000243694.2	2.86 × 10^52^	−1.749	2.666	0.917
TCGA_LIHC	2	*CTC-537E7.3*	ENSG00000248884.1	2.98 × 10^20^	−1.402	2.028	0.626
TCGA_LIHC	3	*RP11-789C1.1*	ENSG00000250266.1	2.59 × 10^11^	−1.148	1.403	0.254
TCGA_LIHC	4	*RP11-252E2.2*	ENSG00000261058.1	5.17 × 10^18^	−0.981	1.455	0.474
TCGA_LIHC	5	*RP11-172E9.2*	ENSG00000230058.1	4.83 × 10^8^	−0.881	1.074	0.193
TCGA_LIHC	6	*RP11-434D9.1*	ENSG00000249364.4	1.87 × 10^10^	−0.869	1.613	0.744
TCGA_LIHC	7	*RP11-119D9.1*	ENSG00000251637.5	3.45 × 10^16^	−0.777	1.693	0.916
TCGA_LIHC	8	*RP11-513G11.3*	ENSG00000238097.1	5.94 × 10^15^	−0.741	1.733	0.992
TCGA_LIHC	9	*FLJ22763*	ENSG00000241224.5	3.59 × 10^17^	−0.681	1.192	0.511
TCGA_LIHC	10	*RP11-327J17.2*	ENSG00000259359.1	1.16 × 10^11^	−0.639	1.35	0.71
GSE77314	1	*LINC01093*	ENSG00000249173.5	3.08 × 10^45^	−4.697	5.232	0.535
GSE77314	2	*RP11-6B4.1*	ENSG00000243694.2	3.14 × 10^23^	−3.023	3.871	0.848
GSE77314	3	*RP11-434D9.1*	ENSG00000249364.5	3.74 × 10^22^	−2.594	3.594	1
GSE77314	4	*FLJ22763*	ENSG00000241224.6	1.87 × 10^22^	−2.379	3.061	0.682
GSE77314	5	*AC016768.1*	ENSG00000232451.1	2.16 × 10^22^	−1.586	2.355	0.769
GSE77314	6	*RP4-568C11.4*	ENSG00000274173.1	2.46 × 10^17^	−1.516	2.109	0.593
GSE77314	7	*RP11-439C15.4*	ENSG00000253764.1	1.02 × 10^22^	−1.49	1.679	0.19
GSE77314	8	*RP11-153K11.3*	ENSG00000233590.1	1.99 × 10^12^	−1.467	1.979	0.512
GSE77314	9	*CTC-537E7.3*	ENSG00000248884.1	5.63 × 10^18^	−1.456	1.762	0.306
GSE77314	10	*RP11-789C1.2*	ENSG00000251061.2	1.60 × 10^14^	−1.439	1.506	0.066
GSE124535	1	*RP11-789C1.1*	ENSG00000250266	4.30 × 10^9^	−1.232	1.555	0.322
GSE124535	2	*CTC-537E7.3*	ENSG00000248884	1.05 × 10^11^	−1.228	1.745	0.517
GSE124535	3	*RP11-290F20.3*	ENSG00000224397	1.09 × 10^9^	−1.096	2.034	0.939
GSE124535	4	*CTC-297N7.9*	ENSG00000264016	2.19 × 10^10^	−0.986	1.947	0.961
GSE124535	5	*RP11-327J17.2*	ENSG00000259359	9.98 × 10^9^	−0.978	1.693	0.715
GSE124535	6	*CTB-167B5.2*	ENSG00000264868	5.36 × 10^10^	−0.873	1.601	0.728
GSE124535	7	*RP11-252E2.2*	ENSG00000261058	1.71 × 10^7^	−0.822	1.344	0.522
GSE124535	8	*RP11-557H15.4*	ENSG00000232310	9.63 × 10^7^	−0.774	1.413	0.639
GSE124535	9	*RP11-863P13.2*	ENSG00000261816	3.03 × 10^5^	−0.722	1.068	0.347
GSE124535	10	*RP11-88H9.2*	ENSG00000231437	4.12 × 10^7^	−0.636	1.135	0.498

## Data Availability

The data supporting the findings of this study are available from the corresponding author upon reasonable request.

## Supplementary Materials

The following supporting information can be downloaded at: https://www.mdpi.com/article/10.3390/cimb47070563/s1.
