# Supplementary material for: CTC-537E7.3 as a Liver-Specific Biomarker for Hepatocellular Carcinoma: Diagnostic and Prognostic Implications
Source: Curr Issues Mol Biol. 2025 Jul 18;47(7):563. doi: 10.3390/cimb47070563 (PMC12293479; doi:10.3390/cimb47070563)
Supplement: Supplementary file 1 [file cimb-47-00563-s001.zip › Supplementary Figures.pdf]

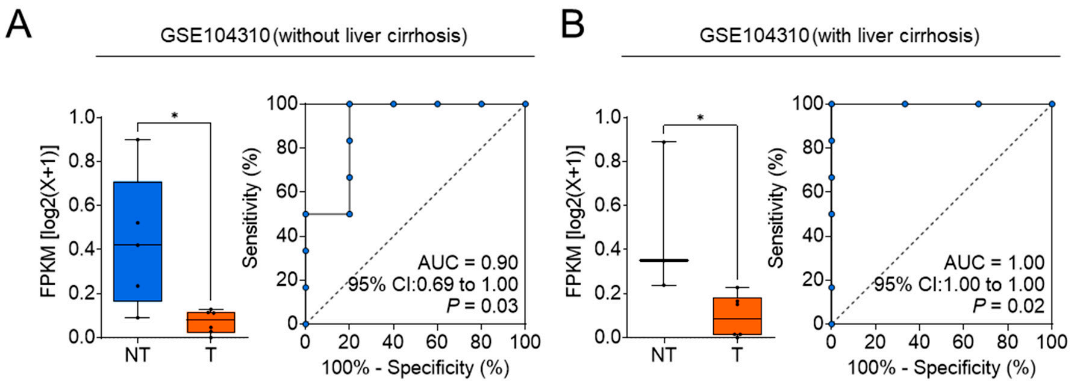

Supplementary Figure S1. Diagnostic performance of *CTC-537E7.3* in the GSE104310 cohort stratified by cirrhosis status. (A) Non-cirrhotic livers; (B) cirrhotic livers. Left panels: box plots of *CTC-537E7.3* expression [$\log_2$(FPKM + 1)] in non-tumor (NT) versus tumor (T) tissue. Right panels: ROC curves illustrating the ability of *CTC-537E7.3* to distinguish HCC from NT, with AUC, 95 % CI and statistical significance was defined as * $P < 0.05$.

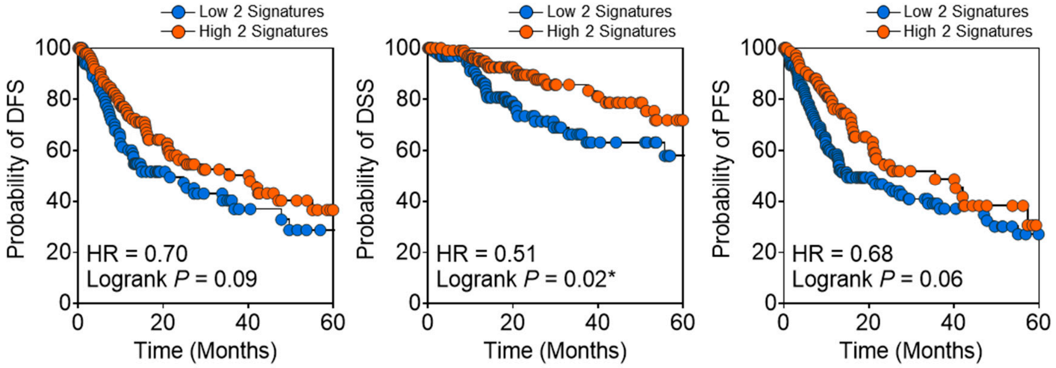

Supplementary Figure S2. Kaplan–Meier analyses of additional clinical endpoints based on the combined CTC-537E7.3/PLGLB1 expression signature. Patients were dichotomized into a concordant high-expression group (High 2 Signatures, orange) and a concordant low-expression group (Low 2 Signatures, blue). Survival curves are shown for disease-free survival (DFS), disease-specific survival (DSS) and progression-free survival (PFS). Hazard ratios (HRs), 95 % confidence intervals (CIs) and log-rank *P*-values are indicated; *P < 0.05.
