# Supplementary material for: CTC-537E7.3 as a Liver-Specific Biomarker for Hepatocellular Carcinoma: Diagnostic and Prognostic Implications
Source: Curr Issues Mol Biol. 2025 Jul 18;47(7):563. doi: 10.3390/cimb47070563 (PMC12293479; doi:10.3390/cimb47070563)
Supplement: Supplementary file 1 [file cimb-47-00563-s001.zip › Supplementary Tables.pdf]

Supplementary Tables

Table S1. Univariate and Multivariate Cox Regression Analyses for Overall Survival, Disease-Free Survival, Disease-Specific Survival, and Progression-Free Survival.

**Univariate Cox Analysis for OS**		
**Variable**	**HR (95% CI)**	***P*-value**
CTC-537E7.3 expression	0.60 (0.37–0.98)	0.041
Sex (male)	0.64 (0.36–1.13)	0.124
Total bilirubin (mg/dL)	1.34 (0.97–1.86)	0.076
Creatinine (mg/dL)	0.83 (0.48–1.43)	0.496
Serum AFP (ng/mL)	1.00 (1.00–1.00)	0.12
Vascular invasion	2.12 (1.19–3.76)	0.011
Histologic Grade	1.82 (1.04–3.19)	0.037
Age	1.05 (1.02–1.07)	0.0006
**Multivariate Cox Analysis for OS**		
**Variable**	**HR (95% CI)**	***P*-value**
CTC-537E7.3 expression	0.589 (0.362 – 0.959)	0.033
Vascular invasion	1.800 (1.027 – 3.156)	0.04
Histologic Grade	1.807 (1.034 – 3.157)	0.038
Age	1.045 (1.020 – 1.070)	0.0004
**Univariate Cox Analysis for DFS**		
**Variable**	**HR (95% CI)**	***P*-value**
CTC-537E7.3 expression	0.73 (0.54–0.99)	0.0408
Sex (male)	0.88 (0.57–1.35)	0.551
Total bilirubin (mg/dL)	1.13 (0.87–1.47)	0.378
Creatinine (mg/dL)	0.95 (0.80–1.12)	0.516
Serum AFP (ng/mL)	1.00 (1.00–1.00)	0.139
Vascular invasion	1.90 (1.26–2.87)	0.00236
Histologic Grade	1.42 (0.95–2.13)	0.0843
Age	0.99 (0.98–1.01)	0.416
**Multivariate Cox Analysis for DFS**		

Variable	HR (95% CI)	*P*-value
CTC-537E7.3 expression	0.762 (0.56–1.04)	0.082
Vascular invasion	1.799 (1.19–2.72)	0.0056
**Univariate Cox Analysis for DSS**		
**Variable**	**HR (95% CI)**	***P*-value**
CTC-537E7.3 expression	0.738 (0.436–1.249)	0.258
Sex (male)	0.659 (0.323–1.344)	0.251
Total bilirubin (mg/dL)	1.090 (0.658–1.806)	0.737
Creatinine (mg/dL)	0.296 (0.079–1.108)	0.071
Serum AFP (ng/mL)	1.000 (1.000–1.000)	0.526
Vascular invasion	1.901 (0.902–4.006)	0.091
Histologic Grade	1.840 (0.910–3.724)	0.09
Age	1.021 (0.993–1.050)	0.144
**Univariate Cox Analysis for PFS**		
**Variable**	**HR (95% CI)**	***P*-value**
CTC-537E7.3 expression	0.712 (0.524–0.968)	0.03
Sex (male)	0.831 (0.544–1.270)	0.393
Total bilirubin (mg/dL)	1.108 (0.847–1.449)	0.453
Creatinine (mg/dL)	0.966 (0.870–1.071)	0.508
Serum AFP (ng/mL)	1.000 (1.000–1.000)	0.148
Vascular invasion	2.000 (1.325–3.006)	0.00094
Histologic Grade	1.379 (0.925–2.056)	0.115
Age	0.994 (0.978–1.009)	0.428
**Multivariate Cox Analysis for PFS**		
**Variable**	**HR (95% CI)**	***P*-value**
CTC-537E7.3 expression	0.75 (0.55–1.02)	0.062
Vascular invasion	1.89 (1.26–2.86)	0.002

OS: Overall Survival, DFS: Disease-Free Survival, DSS: Disease-Specific Survival, PFS: Progression-Free Survival, HR: Hazard Ratio, CI: Confidence Interval, AFP: Alpha-Fetoprotein

Table S2. Top 10 miRNAs with Highest Target Scores for CTC-537E7.3 in miRDB.

Target rank	Target score	miRNA name	CancerExp	NormalExp	Fold change	*P*-value	FDR
1	94	hsa-miR-6875-3p	0.04	0.02	2	0.19	0.45
2	94	hsa-miR-759	0.01	0.01	1	0.71	0.78
3	93	hsa-miR-6072	0.01	0.01	1	0.71	0.78
4	91	hsa-miR-6891-3p	0.02	1.00E-02	1.68E+00	0.27	0.58
**5**	**83**	**hsa-miR-190b-5p**	**1.34**	**0.16**	**8.36**	**5.80E-11**	**1.40E-09**
6	83	hsa-miR-190a-5p	3.43	3.46	0.99	0.075	0.23
7	83	hsa-miR-4765	0.01	0.01	1.2	0.53	0.78
8	79	hsa-miR-5691	0.02	0.02	1.12	0.45	0.78
9	77	hsa-miR-5586-3p	0.02	0.02	1.29	0.68	0.78
10	76	hsa-miR-6805-3p	0.01	0.01	1.32	0.47	0.78

miRNA: microRNA, CancerExp: Cancer Expression, NormalExp: Normal Expression, *P*-value: Probability value, FDR: False Discovery Rate

Table S3. Predicted Target Genes of hsa-miR-190b-5p with Strong Repression Potential Identified by TargetScan (Score ≤ –0.5)

Target gene	Gene name	Cumulative weighted context++ score	Total context++ score
NEUROD1	neuronal differentiation 1	-0.97	-0.97
KPNA5	karyopherin alpha 5 (importin alpha 6)	-0.9	-1.02
ERG	v-ets avian erythroblastosis virus E26 oncogene homolog	-0.73	-0.73
VWC2	von Willebrand factor C domain containing 2	-0.8	-0.8
LPP	LIM domain containing preferred translocation partner in lipoma	-0.52	-0.52
CSRNP3	cysteine-serine-rich nuclear protein 3	-0.51	-0.51
STK38L	serine/threonine kinase 38 like	-0.5	-0.6
GUCY1B3	guanylate cyclase 1, soluble, beta 3	-0.53	-0.53
ASAP2	ArfGAP with SH3 domain, ankyrin repeat and PH domain 2	-0.52	-0.7
EMR3	egf-like module containing, mucin-like, hormone receptor-like 3	-0.64	-0.64
OSBPL6	oxysterol binding protein-like 6	-0.51	-0.62
KLHL42	kelch-like family member 42	-0.53	-0.55
TOMM5	translocase of outer mitochondrial membrane 5 homolog (yeast)	-0.81	-0.82
TMEM161B	transmembrane protein 161B	-0.54	-0.54
PSMA8	proteasome (prosome, macropain) subunit, alpha type, 8	-0.55	-0.55
ARPC5	actin related protein 2/3 complex, subunit 5, 16kDa	-0.69	-0.69
IL2	interleukin 2	-0.69	-0.69
DCUN1D1	DCN1, defective in cullin neddylation 1, domain containing 1	-0.61	-0.66
RFK	riboflavin kinase	-0.56	-1.01
PLGLB1	plasminogen-like B1	-0.56	-0.56
PLGLB2	plasminogen-like B2	-0.56	-0.56
BBS4	Bardet-Biedl syndrome 4	-0.51	-0.51
CNN3	calponin 3, acidic	-0.53	-0.53
CSN2	casein beta	-0.61	-0.61
